# Immunopathogenesis and Therapeutic Implications in Basal Cell Carcinoma: Current Concepts and Future Directions

**DOI:** 10.3390/medicina61111914

**Published:** 2025-10-25

**Authors:** Helen C. Steel, Theresa M. Rossouw, Ronald Anderson, Lindsay Anderson, Daniel van Tonder, Teresa Smit, Bernardo Leon Rapoport

**Affiliations:** 1 Department of Immunology, School of Medicine, Faculty of Health Sciences, University of Pretoria, Prinshof 0083, South Africa; helen.steel@up.ac.za (H.C.S.); theresa.rossouw@up.ac.za (T.M.R.); 2 The Clinical and Translational Research Unit of the Medical Oncology Centre of Rosebank, Saxonwold, Johannesburg 2196, South Africa; ronald.anderson@rosebankoncology-ctru-co.za (R.A.); danielvantonder@rosebankoncology-ctru.co.za (D.v.T.); tsmit@rosebankoncology-ctru.co.za (T.S.); 3 Curo Oncology, Eugene Marais Hospital, Les Marais, Pretoria 0084, South Africa; curo3onc@curo-oncology.co.za

**Keywords:** co-inhibitory immune checkpoints, driver mutations, hedgehog signaling pathway, immune evasion/exclusion, immune suppressive viruses, immunotherapy, regulatory T-cells, transforming growth factor-β1, tumor mutational burden, ultraviolet radiation

## Abstract

This review is focused on understanding the reasons why basal cell carcinoma (BCC), the most common, increasingly prevalent cancer, is classified as an “immune excluded” malignancy. It is, despite manifesting one of the highest tumor mutational burdens of any solid human malignancy, considered to be a biomarker of enhanced tumor immunogenicity and efficacy of tumor-targeted immunotherapy. Following a brief clinical overview, the balance of the review addresses important translational issues based on recent insights into the mechanisms underpinning immune exclusion/evasion in BCC. These include, firstly, the role of infectious agents and non-infectious potential causes of predisposition for and/or exacerbation of disease development and progression. Secondly, an overview of existing and emerging novel therapeutic strategies to ameliorate immune exclusion in BCC based on targeting several key immunosuppressive mechanisms. These are (i) inappropriate activation of the hedgehog signaling pathway (HHSP) due to formation of key driver mutations; (ii) interference with the presentation of tumor-specific antigens/neoantigens to cytotoxic T-cells; (iii) attenuation of the influx of anti-tumor natural killer cells; (iv) the recruitment and activation of immune suppressive regulatory T-cells; and (v) localized and systemic immune dysfunction achieved via elevated levels of soluble co-inhibitory immune checkpoint proteins (ICPs). The final section is focused on current and emerging pharmacologic and immune-based therapies.

## 1. Introduction

Cutaneous basal cell carcinoma (BCC) is the most common cancer in humans [1]. This condition is associated with low mortality; however, morbidity is high due to local invasion of tissue and structures, including muscles and bones.

In recent years, novel therapies and biomarkers have been identified to decrease morbidity and improve the quality of life of these patients. The current review will briefly overview the epidemiology, clinical features, and diagnosis of BCC, as well as risk factors, including exposure to ultraviolet radiation (UVR) and its association with pro-tumorigenic mutagenesis and immune suppression. The next phase of this review manuscript will describe key driver mutations in the pathogenesis of BCC linked predominantly to mutations of key regulatory genes of the hedgehog signaling pathway (HHSP) and other regulatory pathways, as well as key events in immune pathogenesis, specifically those underpinning mechanisms of tumor evasion operative both in the tumor microenvironment (TME) and systemically.

The prominent role of cytokines, particularly transforming growth factor-β1 (TGF-β1), chemokines, adenosine, and regulatory T-cells (Tregs), all implicated as being key players in immune suppression, will be discussed. Additional systemic mediators and biomarkers, such as soluble immune checkpoint proteins (sICPs), including co-inhibitory ICPs, such as programmed cell death protein 1 (PD-1) and its ligand PD-L1, cytotoxic T-cell associated protein 4 (CTLA-4), and lymphocyte-associated gene 3 (LAG-3), will also be reviewed. The final sections of the review will focus on systemic biomarkers with the potential to guide patient management. An update on the immunotherapy of recurrent and advanced disease and the search for potential novel immune-based treatments will also be addressed, as well as key future directions.

The review is focused primarily on the identification of prominent mechanisms and mediators of immune exclusion and evasion operative in BCC and potential therapeutic targets, many of which are unique. This manuscript is an extensive non-systematic narrative literature review, encompassing mostly recent publications, insofar as possible, based on searches of prominent databases, including, but not limited to, PubMed, PMC, and Medline.

## 2. Epidemiology, Clinical Features, Histopathology, Risk Factors, and Diagnosis of BCC

Although the most prominent type of human malignancy, the true prevalence of BCCs is likely to be underestimated, as BCCs are not routinely recorded in cancer registries and are not always confirmed by histology. The lifetime risk of developing BCC is reported as approximately 20% overall and 30% for white individuals [1]. The true incidence of BCC is, however, difficult to determine [2], possibly due to underreporting. Nevertheless, a worldwide increase in BCC is seen, with the incidence in the United States of America reportedly increasing at a rate of 4% to 8% annually. This malignancy is predominantly seen in older individuals and in men (male-to-female ratio of 1.5–2.1) [1].

Ultraviolet radiation exposure via sunlight is the most important risk factor for the development of BCC. Skin pigmentation plays a protective role, with a lower incidence seen in dark skinned individuals. Factors such as fair skin, albinism, red or blonde hair, light-colored eyes, difficulty tanning, and a tendency to develop freckles are individual risk factors for BCC [1]. Several genetic conditions are associated with an increased risk of this condition, including the autosomal dominant nevoid BCC syndrome (NBCCS) (otherwise known as Gorlin syndrome), which results in an aberrant increase in the Sonic HHSP [1].

Basal cell carcinoma typically presents as a slow-growing skin cancer, most commonly in the head and neck region, with some subtypes showing a predilection for the trunk [2]. There is a variety of clinical and histopathological subtypes of BCC, including nodular, superficial, infundibulocystic, fibroepithelial, morpheaform, infiltrative, micronodular, and basosquamous [1]. Nodular lesions are the most common and present as shiny papules or nodules with arborizing telangiectasia. Larger lesions may show central ulceration. Superficial lesions are the second most common subtype and appear erythematous and flat with well-defined rolled borders and central clearing. Morpheaform lesions present as white sclerotic or scar-like plaques with poorly defined borders and are associated with aggressive behavior and are more difficult to treat [1,3]. Basosquamous lesions appear as a facial nodule or plaque with ulceration and may show features of both BCC and invasive squamous cell carcinoma (SCC). Presentation is highly polymorphic, and classification into a specific subtype can be difficult.

Dermoscopy is being increasingly utilized to visualize skin structures that are otherwise not visible without magnification. Its use increases the sensitivity and specificity of the diagnosis of BCC. The most prevalent dermoscopic features of this condition are found to be arborizing telangiectasia and shiny white structures. The histopathological subtypes show variation in the frequency of dermoscopic features, even though no single feature is unique to a specific subtype. Nodular BCC shows mostly arborizing vessels, blue-grey ovoid nest and ulceration, while superficial BCC shows short fine telangiectasia, small erosions and concentric or leaf-like structures, while porcelain white features are seen in the morpheaform [4].

Locally advanced disease with deep infiltration into surrounding tissues occurs without timely detection and treatment. However, metastasis is uncommon and seen in less than 1% of patients [2]. The European Association of Dermato-Oncology classification and staging stratifies BCC into four stages. These are the encompassing stages 1 and 2, advanced stage 3, and metastatic stage 4. Stage 1 refers to easy-to-treat common BCC with a low risk of recurrence, while stages 2, 3, and 4 are considered difficult-to-treat. Cases of BCC can be difficult to treat for a multitude of reasons, including tumor location, subtype, recurrence, and previous treatment [2].

## 3. Roles of Human Papillomavirus and Human Immunodeficiency Virus, as Well as Pre-Existing Immune Suppression

Some earlier studies have indicated a potential link between several types of oncogenic viruses. For the purposes of this review, however, we have focused on human papillomaviruses (HPV) and human immunodeficiency virus (HIV) in the development of BCC [5,6].

### 3.1. Human Papillomavirus

It is proposed that carcinogenesis may be driven by the HPV E6 protein, which inhibits apoptosis by blocking the effects of the B-cell lymphoma-2 (Bcl-2) homologous antagonist killer protein [7]. However, while HPV DNA has been detected in some BCC lesions, definitive evidence remains elusive [5,8,9,10].

### 3.2. Human Immunodeficiency Virus

Individuals with HIV are at an increased risk for certain non-melanoma skin cancers, with the most pronounced effect observed in SCC. The impact on BCC is relatively modest, with studies indicating an increased risk of approximately 2-fold [6,11,12,13], and it appears to manifest at a younger age compared to the general population [14]. While the immune system is crucial in BCC development, and conditions like HIV can disrupt normal immune responses, the risk is not strongly linked to HIV-related immune dysfunction, as measured by CD4+ T-cell count and RNA viral load [11,12,14,15].

As stated above, BCC is generally characterized by slow growth and a low potential for metastasis, which may explain why the impact of HIV is less severe compared to SCC, which can progress more aggressively in immunocompromised states [16]. Although the immune system is vital for tumor suppression, its role in BCC development and progression involves complex interactions with other risk factors, such as UVR exposure, genetic predisposition, and possibly life stressors, rather than serving as a primary direct cause of BCC [17]. Another explanation could be that alterations in the HHSP, a common feature in BCC, are less dependent on immune surveillance.

### 3.3. Pre-Existing Non-Infectious Disease-Associated Immune Suppression

Immunosuppression, as such, is a significant host factor affecting BCC incidence and progression [18], particularly in organ transplant recipients. In this setting, the incidence of this malignancy is about 10 times higher than in the general population [19]. In the context of BCC, the immunological landscape involves key co-inhibitory ICPs, such as PD-1 and its ligand, PD-L1, as well as CTLA-4, which negatively regulate T-cell activity. These co-inhibitory ICPs contribute to immune evasion, and this process is exacerbated in immunocompromised individuals [20]. Unlike SCC, however, there is no strong evidence suggesting that organ transplant recipients experience more aggressive forms of BCC. While some studies have reported the development of lesions at a significantly younger age, with more lesions on extra-cephalic locations and at unusual sites [21], other studies have failed to confirm these findings [22].

In the case of hematological malignancies, patients with non-Hodgkin’s lymphoma (NHL) exhibit an increase in CD14+ HLA-DR^low^-expressing monocytes, which possess immunosuppressive properties, along with elevated levels of CD4+ CD25+ Tregs [23,24]. These alterations have been linked to a higher incidence of BCC. In this context, those living with chronic lymphocytic leukemia (CLL) face a greater risk compared to those with non-CLL NHL [25]. Notably, the clinical progression is more aggressive, and the recurrence rates are significantly higher [25,26]. Accordingly, BCC has been linked to a poorer prognosis in patients with NHL [27,28].

A comprehensive study from the United Kingdom further revealed that patients with BCC are significantly more likely to have a medical history of rheumatoid arthritis (RA) or inflammatory bowel disease (IBD), attributable to either iatrogenic or non-iatrogenic immunosuppression [18]. Beyond the specific photosensitizing and oncogenic effects of certain immunosuppressive drugs, it is believed that impaired immune surveillance in this context allows for the unchecked growth of cancer-initiated cells [29].

## 4. Driver Mutations/Mechanisms in BCC

Notably, of all types of cancer, BCC is characterized by the highest gene mutation rate, with over 65 mutations/megabase pair (Mbp) being identified [30,31]. The identification and understanding of these driver mutations of BCC have led directly to the development of targeted treatments and better management of BCC. Below, the genes that have been identified as driving the pathogenesis of BCC are briefly discussed and summarized in Table 1.

### 4.1. Hedgehog Signaling Pathway

The HHSP, encompassing the Sonic, Indian, and Desert hedgehog (HH) signaling ligands, the cell surface transmembrane receptor Patched (PTCH) (comprising the two homologs PTCH1 and PTCH2), and three glioma-associated (GLI) transcription factors (GLI1, GLI2, and GLI3), is involved in regulating normal cell development, proliferation, and survival. Localized to the primary cilium, the HHSP is largely inactive in the adult; however, it plays an important role in wound healing and tissue repair [32]. This microtubule-based organelle protrudes from the plasma membrane in most cell types, where it detects extracellular signals [33]. Under normal conditions, in the absence of a HH signaling ligand, PTCH1 inhibits Smoothened (SMO), a signaling receptor downstream of PTCH1. This, in turn, prevents the activation of GLI1 and the translocation of this transcription factor to the nucleus, thereby suppressing genes promoting cell growth. When a signaling ligand binds, PTCH1 is inactivated and, as such, SMO is no longer inhibited and binds to the Suppressor of Fused (SUFU) homolog, leading to activation and nuclear translocation of GLI with the subsequent expression of genes leading to the promotion of cell growth and survival [34]. Hyperactivation of the HHSP has been associated with tumor initiation and progression and drug resistance in various types of cancers [35,36,37].

Indeed, mutations in genes involved in the activation of HHP signaling have been demonstrated to play critical roles as the primary drivers of BCC, as elegantly depicted by Bakshi et al. [35]. DNA damage resulting from exposure to UVB leads to mutations in the tumor suppressor gene *PTCH*, which encodes for the cell surface transmembrane receptor [38]. Importantly, *PTCH1* has been identified as the most frequently mutated gene in BCC, leading to more than 70 percent of sporadic BCC cases [30].

Mutations of *PTCH1* inhibit the repressor function of PTCH on SMO, leading to hyperactivation of the Sonic HHSP. Furthermore, *SMO*, which encodes for the signaling receptor, has also been found to be mutated in 10 to 20 percent of BCCs [30,39]. This mutation leads to a ‘gain-of-function’ of the HHSP, despite the inhibitory function of PTCH1. In addition, constitutively activated mutants of *SMO* inhibit the accumulation of the tumor suppressor protein, p53. Inhibition of this protein leads to loss of cellular senescence and apoptosis. Therefore, mutations of *PTCH1* and *SMO* are responsible for the constitutive up-regulation of the HHSP, leading to dysregulation of cell growth and immunosuppression, which, in turn, leads to the promotion and pathogenesis of BCC [30,39]. Mutations affecting SUFU, a negative regulator of GLI activity, although less common, have also been identified as playing a role in BCC and account for approximately eight percent of BCCs [30]. However, these mutations are associated more commonly with NBCCS.

### 4.2. Tp53 Gene Mutations

Another frequently mutated gene associated with the pathogenesis of BCC and other non-melanoma skin cancers is *Tp53*, which encodes for the p53 protein. The p53 protein is responsible for signaling the apoptosis of cells in which DNA damage cannot be repaired, thereby preventing these cells from dividing and differentiating [40]. Mutations in the p53 protein not only result in the loss of tumor suppressor activity and anti-apoptotic function but also lead to the promotion of tumor cell proliferation, angiogenesis, and metastasis [41,42]. The ‘loss-of-function’ of this tumor suppressor gene is reported in between 40 and 65 percent of cases of BCC and is specifically linked to UVB-damage [43].

### 4.3. The Hippo/YAP Signaling Pathway

The Hippo/YAP pathway is a signaling pathway that is associated with controlling organ size, tissue homeostasis, regeneration, and tumorigenesis. This is achieved by regulating cell proliferation and apoptosis. The Hippo/YAP pathway is comprised of a cascade of kinases (mammalian STE20-like protein kinase 1/2 (MST1/2) and large tumor suppressor 1/2 (LATS1/2)), which inhibit nuclear translocation of the co-transcriptional factors Yes-associated protein 1/transcriptional co-activator with PDZ-binding motif [also known as WW domain containing transcription regulator 1 (WWTR1)] (YAP/TAZ) and its association with the transcriptional-enhanced associated domain (TEAD) [44].

This pathway has been reported to be involved in the tumorigenesis and metastasis of several types of cancer [45]. Indeed, albeit in a murine model, YAP/TAZ has recently been found to be required for the development of BCC triggered by hyperactivation of the HHSP [46]. These authors also demonstrated that BCC initiation could be prevented by the deletion of YAP and TAZ. In humans, YAP/TAZ has reportedly been found in the nucleus of BCC cells in over 90 percent of individuals presenting with this form of cancer, further substantiating the role of YAP/TAZ in the initiation of BCC [46].

Treatments that focus on Hippo signaling, which acts as a tumor suppressor pathway, and YAP/TAZ-associated proteins are attractive targets for BCC and other skin-associated cancer therapies. A number of studies targeting this pathway are currently being conducted with several therapeutic candidates showing promise in clinical trials as described below [47].

### 4.4. Other Mechanisms Driving the Pathogenesis of BCC

Telomeres, the terminal ends of chromosomes, shorten (by 30 to 200 bp) with each cell division, and cellular senescence and apoptosis are initiated when telomeres shorten to a critical length [48]. A high prevalence of mutations of the *telomerase reverse transcriptase* (*TERT*) promoter (up to 56 percent) has been reported in BCC [49,50,51]. These ‘UV-signature’ mutations maintain telomere length and genomic stability through increased expression of telomerase, thus allowing tumor cells to continuously divide, avoiding senescence or apoptosis [50,52].

UVB-associated mutations in the promoter region of the *diphthamide biosynthesis protein 3* (*DPH3*) and *oxidoreductase NAD-binding domain containing 1* (*OXNAD1*) genes are also found in BCC, with mutations involving *DPH3* reported to occur at a frequency of 42 percent [51]. These mutations prevent the binding of E26 transformation-specific (Ets) transcription factors, which play important roles in the proliferation, differentiation, and apoptosis of cells, as well as in the remodeling of tissue [53].

The gene locus of the transcription factor, MYCN, associated with cell growth and differentiation, has also been identified as a driver mutation in BCC [54]. This transcription factor functions as a downstream effector in the Sonic HHSP. Importantly, it has been reported that increased expression of MYCN is associated with more aggressive subtypes of BCC [54]. Combined therapeutic agents, targeting both the HHSP and MYCN, may therefore improve the treatment of BCC and warrant further investigation [55].

Micro RNAs (miRNAs) are small, non-coding RNA molecules that regulate post-transcriptional gene expression by ‘silencing’ the gene. They play an important role in the development, maturation, differentiation, and apoptosis of the cell. In addition, miRNAs are involved with cell signaling, cellular interactions, and homeostasis [56,57]. Due to the ability of miRNAs to alter cellular pathways through their interaction with target genes, miRNAs are found to be dysregulated in many disease states, including cancer, with BCC being no exception [58]. Tumor-associated miRNAs are highly expressed, while expression of suppressor miRNAs is down-regulated. Importantly, along with various other types of cancer, BCC has been found to actively secrete miRNAs into the bloodstream, underscoring the potential value of miRNAs as non-invasive targets as diagnostic markers, as well as having therapeutic potential [59,60,61]. Modifying or reversing changes in miRNA expression form the basis of miRNA therapeutics, as recently reviewed by Diener et al. [62]. Importantly, regulation of the HHSP by miRNAs has been implicated in mediating the resistance of certain cancer cells to chemotherapeutic drugs, an observation that needs to be investigated in the setting of BCC [63]. It is also noteworthy, however, that the effects of this otherwise highly efficient class of treatments are not restricted to the target tissues or cells and can cause systemic side effects.

**Table 1:** Summary of the main drivers of basal cell carcinoma pathogenesis and the mechanisms involved.

Driver of BCC Pathogenesis	Protein Involved	Mechanism	References
*PTCH1*	PTCH1 receptor	No inhibition of SMO leading to hyperactivation of the HHSP.	[30,39]
*SMO*	G-coupled SMO receptor	‘Gain of function’ despite PTCH1 inhibitor function leading to hyperactivation of HHSP.	[30,39]
*SMO*	G-coupled SMO receptor	Inhibits accumulation of the tumor suppressor protein (p53), leading to loss of cellular senescence and apoptosis.	[30,39]
*SUFU*	SUFU homolog	Activation and nuclear translocation of glioma- associated (GLI) transcription factor 1 leading to cell growth and survival.	[30,34]
*Tp53*	p53	Loss of tumor suppressor function leading to promotion of proliferation, angiogenesis, and metastasis.	[41,42]
LATS1/2 and MST1/2	Tumor suppressor kinases	Dephosphorylation results in translocation of YAP/TAZ to the nucleus, where it associates with TEAD, leading to tumor proliferation and survival.	[45]
*TERT*	Telomerase	Maintains telomere length and genomic stability through increased expression of telomerase, allowing cells to continuously divide, avoiding senescence or apoptosis.	[52]
*DPH3* and *OXNAD1*	DPH3 and OXNAD1 proteins	Prevent the binding of E26 transformation-specific transcription factors leading to proliferation and differentiation of cells.	[51,53]
*MYCN*	Transcription factor	Downstream effector in the Sonic HHSP.	[54]
MiRNAs	Non-coding RNA molecules	Interact with target genes and alter cellular pathways.	[58]

Abbreviations: DPH3, diphthamide biosynthesis protein 3; GLI, glioma-associated; HHSP, hedgehog signaling pathway; LATS1/2, large tumor suppressor 1/2; MST1/2, mammalian STE20-like protein kinase 1/2; MiRNAs, micro RNAs; OXNAD1, oxidoreductase NAD-binding domain containing 1; p53, tumor suppressor protein; PTCH1, patched 1; SMO, smoothened; SUFU, suppressor of fused; TAZ, transcriptional co-activator with PDZ-binding motif; TEAD, transcriptional-enhanced associated domain; TERT, telomerase reverse transcriptase; YAP, yes-associated protein 1.

## 5. Immune Landscape of Basal Cell Carcinoma

Data derived from several studies involving cell lines and murine models of experimental tumorigenesis, as well as an analysis of isolated tumor tissue, have demonstrated that BCC cells, which originate from hair follicle stem cells, differentiate along hair cell follicle lineages [64,65,66].

From an immunological perspective, BCC is an enigmatic type of human, solid malignancy, which, despite manifesting a high tumor mutational burden (TMB) (as described above), which is among the highest of any solid human cancers, displays poor immunogenicity due to restricted access of anti-tumor immune mechanisms (as described below). This is characterized by minimal infiltration of protective, anti-tumor cytotoxic T-cells and natural killer (NK) cells into tumor nests, amongst other mechanisms of immune evasion, to failure of presentation of novel, mutated tumor antigens [67,68].

### The Tumor Mutational Burden

The TMB is a positive, albeit indirect, indicator of tumor immunogenicity, which predicts survival in immunotherapy-naïve cancer patients, independently of the type of malignancy and clinical stage [69]. It is also recognized as a predictive “agnostic” biomarker of prediction of outcome of immunotherapy in various types of solid malignancy [70]. Paradoxically, as mentioned above, BCC with its high TMB, is, however, a notable exception, demonstrating absent/weak responses to neoantigens derived from UVR-mutated somatic genes, resulting in variable responses to immunotherapy.

In this context, several studies have attributed the low immunogenicity of BCC to the failure of tumor cell expression of major histocompatibility class I (MHC-1) molecules, which are essential for presentation of tumor neoantigens to cytotoxic T-cells as described below.

The resultant paucity of cytotoxic T-cells in proximity to tumor cell nests contrasts with the peritumoral zone, which is substantially populated by immune suppressive Tregs, comprised of various cellular subtypes with different mechanisms of pro-tumorigenic activity [71,72].

## 6. Mechanisms of Immune Evasion/Exclusion

The following section summarizes several prominent mechanisms of immune evasion operative in BCC, which contribute to sustaining a state of immune exclusion and tumor persistence.

### 6.1. Interference with the Presentation of Tumor Antigens/Neoantigens to Cytotoxic T-Cells

Dysregulation of presentation of tumor antigens/neoantigens is a significant mechanism of failure of recruitment/activation of anti-tumor cytotoxic T-cells in BCC, which is a consequence of down-regulation of MHC-1 and/or its key structural component, β2 microglobulin (β2M) [73,74], key mediators of antigen presentation. However, it is only very recently that the molecular mechanisms underpinning the failure of expression of MHC-1 have been identified, creating an opportunity for the development of novel immunotherapeutic strategies.

In this context, Oka et al., while confirming low-level immunogenicity due to defective expression of MHC-1 and β2M in human primary nodular BCC, also observed increased expression of the transcription factor, Forkhead box c1 (Foxc1) in a BCC cell line (UW-BCC1) [66]. Interestingly, Foxc1, which is primarily an inducer of latency in hair follicle stem cells, was found to promote interferon regulatory factor 1-dependent downregulation of genes involved in antigen presentation [66]. This latter activity was achieved at the epigenomic level, via histone deacetylation of these genes, and was attenuated by entinostat, an inhibitor of histone deacetylase. Importantly, topical application of entinostat in combination with the Toll-like 7 receptor agonist, imiquimod, protected mice against the development of BCC in a T-cell-dependent manner, following sequential topical exposure to the chemical mutagen 7,12-dimethylbenz(a)anthracene and UVB [66]. The authors concluded that investigation of “the efficacy of the topical entinostat plus imiquimod combination for BCC treatment in humans” is warranted [66].

### 6.2. Inactivation of Natural Killer Cells by BCC-Derived Soluble CD200

Expression of the immunosuppressive type 1 transmembrane glycoprotein, CD200, also known as OX-2 membrane glycoprotein, has been described in various types of cancer stem cells [75], including BCC, in which expression is restricted to a small population of cancer stem cells [75,76]. Following proteolysis by matrix metalloproteinases 3 and 11, the cleaved variant of CD200 is released in a soluble form (sCD200) into the tumor microenvironment (TME) and systemic circulation. In these environments, sCD200 retains its bioactive, immunosuppressive properties via interaction with the CD200 receptor expressed on NK cells, as well as T-cells [76]. In the case of NK cells, this interaction results in the attenuation of anti-tumor activity by two mechanisms: firstly, suppression of the production of the tumor-inhibitory cytokine, interferon-γ (IFN-γ), and, secondly, by induction of NK cell apoptosis [76].

Although sCD200 is clearly a potentially broad-spectrum immunotherapeutic target, we are currently not aware of the existence of proven, clinically effective antagonists of the immunosuppressive activity of this molecule.

### 6.3. Tregs as Mediators of Immune Evasion in BCC

As mentioned above, Tregs comprise a substantial cellular population of the peritumoral zone in BCC [71], where they orchestrate tumor immune evasion. Accumulation of Tregs is driven by the chemokine C-C motif chemokine 22 (CCL22) produced predominantly by the tumor, as well as by tumor-associated macrophages and dendritic cells in the TME via interaction with its counter receptor, chemokine receptor 4 (CCR4), expressed on Tregs [77]. Additional mechanisms include the interactions of the immunosuppressive cytokine, transforming growth factor-β1 (TGF-β1), and the prostanoid, prostaglandin E2 (PGE2), both of which abound in the TME, with conventional CD4+ T-cells; this interaction results in the transition of these cells to FoxP3+-expressing Tregs [78]. Soluble mediators of immune suppression released by activated Tregs include interleukin (IL)-10, autocrine release of both TGF-β1 and PGE2, the nucleoside, adenosine, and the soluble variants of the co-inhibitory ICPs, CTLA-4 and PD-1 [71,72,78,79].

Potential, albeit unproven, Treg-targeted immunotherapeutic strategies include the development of small-molecule antagonists of CCR4 [80] and, somewhat speculatively, intra-lesional administration of stabilized adenosine deaminase, an adenosine-neutralizing enzyme.

On a more positive note, a novel biological agent, STP705, which is a nanoparticle-packaged preparation, containing a combination of small interfering RNAs targeting TGF-β1 and cyclooxygenase 2 (PGE2 synthesizing enzyme), designed for intra-lesional administration, has shown considerable promise in the treatment of BCC, possibly because this tumor rarely metastasizes [81].

### 6.4. Co-Inhibitory Immune Checkpoint Proteins as Therapeutic Targets in BCC

We have recently demonstrated significantly increased systemic levels of the soluble variants of the co-inhibitory ICPs, CTLA-4, PD-1, and its ligand PD-L1, LAG-3, and TIM-3 (T-cell immunoglobulin and mucin domain-containing protein 3) in patients with BCC, a finding which is consistent with the involvement of these checkpoint proteins in promoting both systemic and intra-tumoral immune suppression in this malignancy [82]. The current status of therapeutic targeting of co-inhibitory ICPs in BCC is presented in the following section of this review.

These various mechanisms, which drive immune exclusion and immune evasion in BCC, as well as targets for immune-based therapies, are summarized below in Table 2.

## 7. Role of Radiation Therapy and Topical Therapies in Basal Cell Carcinoma

While surgical excision with clear margins remains the standard of care for BCCs, alternative treatments are necessary for patients who are poor surgical candidates or where surgery could lead to undesirable cosmetic outcomes [83].

Radiation therapy (RT) has proven to be an effective, non-invasive alternative to surgery, particularly for the elderly or where there is a concern about poor cosmetic outcomes with primary surgical management. In addition, for patients who have declined surgery, RT is an appropriate alternative definitive treatment option for early stage or locally advanced disease [84].

Radiation therapy can be delivered with superficial X-rays, electrons, or photons, depending on the depth of the BCC being treated. Various radiation doses and fractionation schedules have proven to be effective, and specific recommendations are based on numerous factors, including the following [85]:

- Size and site of the BCC
- Lymph node involvement
- Perineural invasion
- Margin status in the adjuvant setting.

Less frequently, brachytherapy can be used for selected cases of superficial, small, and well-defined BCCs, particularly in cosmetically sensitive areas.

Tumor control rates of 90–95% have been reported for primary BCCs treated with RT alone, but variables including histological subtype, tumor size and location, and depth of invasion can influence tumor control rates. When used in the recurrent or salvage setting, tumor control rates drop to between 60 and 90% [86].

In the post-operative setting, possible indications for adjuvant radiation include high-risk features such as close or positive surgical margins (where re-excision is not possible or would result in significant morbidity), perineural invasion, deeply invasive or locally advanced tumors, and recurrent BCCs [85].

The role of radiation should be discussed on a case-by-case basis and is generally not considered to be first-line treatment for younger patients and in low-risk cases due to the risk of long-term toxicity.

Superficial therapies, including topical imiquimod, topical 5-fluorouracil (5-FU), and photodynamic therapy, can be considered in selected cases of superficial BCCs where surgery and RT are impractical or contraindicated. Control rates are approximately 10% lower with these treatment modalities than for surgical excision or conventional RT procedures [87,88].

## 8. Treatment of Patients with Locally Advanced Unresectable or Metastatic BCC

There are two systemic treatment options available for patients with locally advanced unresectable or metastatic disease, which are not amenable to surgery or RT. These options include HHSP inhibitors and immune checkpoint inhibitors with PD-1-targeted monoclonal antibodies (mAbs) [89].

### 8.1. Hedgehog Signaling Pathway Inhibitors

BCC is strongly driven by abnormal activation of the HHSP via *PTCH1* or *SMO* gene mutations [90]. As stated above, *PTCH1* is a tumor suppressor gene encoding the PTCH1 receptor, which, under normal circumstances, keeps the HHSP inactive when there is no ligand (Sonic Hedgehog, SHH) binding to it. PTCH1 is the normal gatekeeper of the HHSP. When SHH is absent, PTCH1 inhibits SMO, blocking downstream activation [39,91]. When SHH binds to PTCH1, this inhibition is no longer present, and SMO becomes active; therefore, cell proliferation is signaled via the triggering of GLI transcription factors. This oncogene has a primarily activating function.

Mutations of *PTCH1* are the most common genetic abnormalities present in BCC patients (70–90%). Mutated PTCH1 cannot inhibit SMO, such that the HHSP stays permanently ‘active’, driving uncontrolled cell growth [39]. Inhibitors of SMO are highly effective in managing advanced BCC, including the orally administered SMO inhibitors vismodegib and sonidegib. Vismodegib is administered orally at a dose of 150 mg once daily. The pivotal phase II clinical trials of ERIVANCE and BOLT reported the long-term efficacy and safety profiles of vismodegib and sonidegib in BCC. The vismodegib response rate was approximately 45–60% in locally advanced BCC and around 30% in metastatic BCC. Common toxicities reported include muscle cramps, alopecia, dysgeusia, fatigue, and weight loss. Sonidegib, the second SMO inhibitor, given at a dose of 200 mg orally once daily, was associated with a similar activity and toxicity profile to vismodegib. Typically, these agents are administered in the frontline setting and are continued until disease progression or severe toxicity occurs [92].

### 8.2. Immune Checkpoint Inhibition with PD-1-Targeted Monoclonal Antibodies

For patients who fail treatment or become intolerant to HHSP inhibitors, ICP blockade becomes crucial in managing BCC [93]. The co-inhibitory ICP molecule, PD-1, is overexpressed on T-cells; when PD-1 binds to its ligand, PD-L1, on tumor cells, the T-cell is ‘switched off’, allowing the tumor to evade the immune system. Additionally, BCCs have a high TMB from UV damage. High TMB is associated with high levels of neoantigens. High TMB cancers respond well to PD-1/PD-L1 blockade, possibly unleashing immune responses to suppressed presentation of tumor neoantigens in BCC [94].

**Figure 1:**
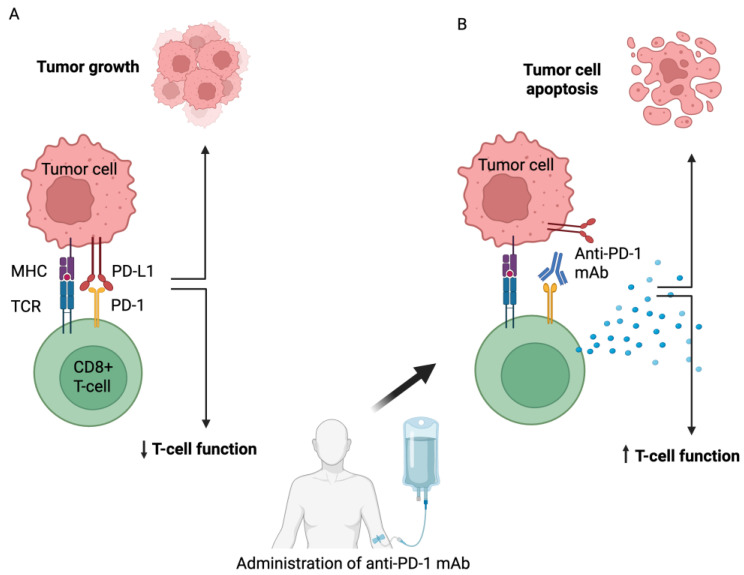
Mechanism of action of anti-PD-1 monoclonal antibodies. Created using Biorender. (**A**) Activated CD8+ T-cells in tumor tissue overexpress the co-inhibitory immune checkpoint protein, PD-1. Binding PD-1 to its ligand, PD-L1, on tumor cells downregulates (↓) T cell activity, leading to tumor growth. (**B**) Administration of the anti-PD-1 monoclonal antibody, cemiplimab, makes PD-1 unavailable for binding to its ligand, thereby increasing (↑) the cytotoxic ability of CD8+ T-cells to induce apoptosis of tumor cells through the release of granzyme and perforin.

Cemiplimab (Libtayo^®^) is an anti-PD-1 mAb approved for patients with locally advanced or metastatic BCC after failure of HHSP inhibitor-based therapy, or intolerance to these agents [95]. The US and European regulatory agencies, the FDA and the EMA, approved this agent in 2021. Cemiplimab is given at a dose of 350 mg intravenously every three weeks until disease progression or unacceptable toxicity becomes evident. The pivotal trial demonstrated a 31% objective response rate for patients with locally advanced disease and approximately 20% for those with metastatic disease (6% complete response and 25% partial response) [96]. Grade 3 and 4 treatment immune-related adverse events (irAEs) occurred in 48% of these patients [96]. These responses are often durable [97,98]. Pembrolizumab (Keytruda) and nivolumab (Opdivo) are not approved for BCC; however, case reports and small series suggest activity, especially in those with high TMB or high microsatellite instability (MSI) tumors [99]. The mechanism of action of cemiplimab in blocking PD-1/PD-L1 interactions is shown in Figure 1.

Immune-related adverse events associated with administration of PD-1-targeted mAbs primarily include dermatological (skin rash and vitiligo), gastroenterological (colitis and diarrhea), and endocrine (thyroiditis, hypophysitis, and adrenal insufficiency) manifestations. Hepatitis and pneumonitis are less commonly encountered irAEs. These toxicities can be severe or even life-threatening [100].

The agents described above, as well as those currently being investigated in clinical trials, are summarized in Table 3.

## 9. Conclusions and Future Directions

Major progress has been made in understanding the immunopathogenesis of BCC, revealing the existence of novel diagnostic and therapeutic biomarkers. From the treatment perspective, future studies should address the clinical utility of HHSP inhibitors in combination with PD-1-targeted mAbs, as well as the use of combinations of immune checkpoint inhibitors, such as anti-PD-1, anti-CTLA-4, and anti-LAG-3, in refractory cases. In this context, combination treatments may increase the risk of systemic toxicities, including irAEs. Current therapeutic gaps include the identification of reliable biomarkers and mechanistic insights into drug resistance mechanisms. Novel HHSP inhibitors should be developed to treat refractory or resistant mutations. Additionally, these agents should be investigated in the neo-adjuvant and high-risk adjuvant settings. Moreover, future studies should investigate biomarkers of immunogenic cell death as indicators of those patients who may benefit from RT.

## Figures and Tables

**Table 2:** Mechanisms and mediators of immune exclusion/evasion operative in BCC and potential therapeutic targets.

Mechanisms	Mediators/Targets	Potential Therapies	References
Failure of presentation of tumor antigens/neoantigens.	Foxc1-mediated histone deacetylation of MHC-1 gene promoter regions.	Inhibition of histone deacetylase by agents such as entinostat.	[66,73,74]
Inactivation of NK cell-mediated anti-tumor activity.	MMP-mediated release of sCD200 from tumor cells, which via interaction with CD200Rs promotes decreased release of IFN-γ and induction of apoptosis.	Seemingly none yet available, but mAb targeting of sCD200 is a possibility.	[75,76]
Accumulation of Tregs in the peritumoral region.	Infiltration of Tregs mediated by the tumor-derived chemokine, CCL22, as well as by exposure of conventional CD4+ T-cells to tumor-derived TGF-β1 and PGE2.	Targeting of TGF-β1 and cyclooxygenase 2 with STP705, as well as possible future targeting of the CCL22 receptor, CCR4, on Tregs with small molecule receptor antagonists.	[77,78,80,81]
Tumor-driven increased expression of co-inhibitory immune checkpoint proteins and release of their bioactive, soluble variants.	CTLA-4, LAG-3, PD-1, PD-L1, TIM-3.	mAb targeting of both the cell-associated and soluble variants of these co-inhibitory immune checkpoint proteins.	[82]

Abbreviations: CCL2, C-C motif chemokine 22; CCR4, chemokine receptor 4; CD200R, cellular receptor for CD200; sCD200, soluble variant of CD200; CTLA-4, cytotoxic T-lymphocyte associated protein 4; Foxc1, Forkhead box c1; IFN-γ, interferon-γ; LAG-3, lymphocyte activation gene 3; mAb, monoclonal antibody; NK, natural killer; MHC-1, major histocompatibility complex class I; MMP, matrix metalloproteinase; PD-1, programmed cell death protein 1; PD-L1, PD-1 ligand; PGE2, prostaglandin E2; TGF-β1, transforming growth factor-β1; TIM-3, T-cell immunoglobulin and mucin domain-containing protein 3; Treg, regulatory T-cell.

**Table 3:** Registered and investigational targeted therapies and immune checkpoint inhibitors used in the treatment of BCC.

**Therapeutic Agent**	**Target**	**Mechanism of Action**	**References**
Vismodegib	HHSP	Competitive inhibitor of SMO.	[101,102]
Sonidegib	HHSP	Selective antagonist of the SMO receptor.	[102,103]
Itraconazole/ Posoconazole	HHSP	Prevents translocation of SMO to the cilium.	[104]
Cemiplimab	PD-1 receptors	Inhibits the binding of PD-1 to PD-L1, thereby enhancing anti-tumor T-cell responses.	[96,105]
Nivolumab	PD-1 receptors	Inhibits the binding of PD-1 to PD-L1, thereby enhancing anti-tumor T-cell responses.	[105,106]
Pembrolizumab	PD-1 receptors	Inhibits the binding of PD-1 to PD-L1, thereby enhancing anti-tumor T-cell responses.	[105,107]
**Agents being assessed in** **clinical trials**			
Silmitasertib	HHSP	Inhibits casein kinase at the terminal end of HP signaling.	[108]
Relatlimab	LAG-3 inhibitor	Inhibits the binding of LAG-3 to its ligands, thereby enhancing anti-tumor T-cell responses.	[109,110]
Ipilimumab	CTLA-4 inhibitor	Inhibits the binding of CTLA-4 to CD80/CD86, thereby enhancing anti-tumor T-cell responses.	[109,111]

Abbreviations: CD, cluster of differentiation; CTLA-4, cytotoxic T-lymphocyte-associated protein 4; HHSP, hedgehog signaling pathway; LAG-3, lymphocyte activation gene 3; PD-1, programmed cell death protein 1; PD-L1, programmed death-ligand 1; SMO, smoothened.

## Data Availability

No new data were created or analyzed in this study.
